# The paradoxical GH response at OGTT does not predict Pasireotide efficacy but matters for glucose metabolism

**DOI:** 10.1007/s40618-025-02534-3

**Published:** 2025-01-22

**Authors:** G. Occhi, G. Voltan, S. Chiloiro, A. Bianchi, P. Maffei, F. Dassie, G. Mantovani, G. Del Sindaco, D. Ferone, F. Gatto, M. Losa, S. Cannavò, C. Scaroni, F. Ceccato, On behalf of the Pituitary Club of the Italian Society of Endocrinology

**Affiliations:** 1 Department of Biology, University of Padova, Via Ugo Bassi 58/B, 35128 Padua, Italy; 2 Department of Medicine (DIMED), University of Padova, Padua, Italy; 3 Endocrine Disease Unit, University-Hospital of Padova, Padua, Italy; 4 Pituitary Unit, Division of Endocrinology and Metabolism, IRCCS Fondazione Policlinico Universitario A. Gemelli, Rome, Italy; 5 Department of Translational Medicine and Surgery, University Cattolica del Sacro Cuore, Rome, Italy; 6 Department of Clinical Sciences and Community Health, University of Milan, 20122 Milan, Italy; 7 Endocrinology Unit, Fondazione IRCCS Ca’ Granda Ospedale Maggiore Policlinico, 20122 Milan, Italy; 8 Endocrinology Unit, Department of Internal Medicine, IRCCS Ospedale Policlinico San Martino, Genoa, Italy; 9 Department of Neurosurgery, IRCCS San Raffaele Scientific Institute, Vita-Salute University, Milan, Italy; 10 Department of Human Pathology in Adulthood and Childhood “G. Barresi”, University of Messina, Messina, Italy

**Keywords:** Acromegaly, GH profile, Oral glucose tolerance test, Pasireotide, Glucose metabolism alterations

## Abstract

**Purpose:**

A paradoxical increase in GH after oral glucose load (GH-Par) characterizes about one-third of acromegaly patients and is associated with a better response to first-generation somatostatin receptor ligands (fg-SRLs). Pasireotide is typically considered as a second-/third-line treatment. Here, we investigated the predictive role of GH-Par in pasireotide response and adverse event development.

**Methods:**

we collected a multicenter Italian retrospective cohort of 59 patients treated with pasireotide for at least 3 months, all having GH profile from OGTT. IGF-1 normalization or at least 30% reduction at the last follow-up visit defined a responder patient.

**Results:**

Considering the entire cohort, median IGF-1 levels before pasireotide (available in 57 patients) were 1.38 times the upper limit of normal (ULN) in patients with large (median size 18 mm) and invasive (82%) adenomas after failure of fg-SRL treatment. After a 40-month median treatment, pasireotide effectively reduced IGF-1 ULN levels in 41 patients, 37 of whom achieving normalization, and 4 with a ≥ 30% reduction. Thirteen patients were classified as GH-Par. The median pasireotide duration, dosage, and efficacy (9/12 responder in the GH-Par group and 32/45 in the GH-NPar) were similar between groups. However, the occurrence of new-onset or worsening glucose metabolism alterations (GMAs) after pasireotide was more frequent in GH-NPar (from 37 to 80%; p < 0.001) compared to GH-Par patients (from 69 to 76%), likely due to the higher prevalence of pre-existing GMAs in the GH-Par group before starting pasireotide (p = 0.038).

**Conclusions:**

The GH-Par does not predict the response to pasireotide in acromegaly but can predict a worse metabolic profile.

## Introduction

Somatostatin receptor ligands (SRLs) are effectively used in the management of acromegaly [1–3]. According to their development and clinical use, they are categorized into first-generation SRLs (fg-SRLs), namely octreotide (OCT) and lanreotide (LAN), and second-generation SRLs, specifically pasireotide (PAS). The most significant difference among them lies in their receptor affinity: while the binding to somatostatin (SST) receptor type-2 is common to all SRLs, PAS exhibits a higher affinity for SSTR5 [4]. In the acromegaly treatment flowchart, PAS is recommended as a second- or third-line treatment in case of resistance to fg-SRLs, persistent GH or IGF-1 excess after surgery, or as a bridge treatment while awaiting the effects of radiotherapy [1, 5]. Its efficacy in the normalization of GH and IGF-1 levels has been proved in randomized and extension trials [6–8], and dose up-titration enriched the number of responders, suggesting that the PAS effect may be both time- and dose-dependent. Regarding its ability in eliciting the shrinkage of GH-secreting pituitary adenomas (GH-sec PAs), PAS has demonstrated comparable efficacy to that of fg-SRLs [9], where a substantial reduction in adenoma size has been documented in about 50% of cases [10]. On the other hand, glucose metabolism alterations (GMAs) typically observed in acromegaly are potentially exacerbated by PAS treatment. Hyperglycemia has been reported in 40–70% of patients treated with PAS [6–8], a significantly higher rate compared to those treated with fg-SRLs, which induce moderate effects on glucose metabolism (any significant change in patients without diabetes and a slight worsening of metabolic control in up to one-quarter of patients with diabetes [11]).

Considering predictive factors of response to medical therapy in acromegaly, several clinical, radiological, and histological features have been proposed and validated for fg-SRLs [12]. Responsive acromegaly patients, often have smaller intra-sellar tumors, with lower GH and IGF-1 levels [13] and a low proliferation rate [14]. Densely granulated adenomas [15, 16] and adenomas with a hypointense signal in T2-weighted magnetic resonance imaging (MRI) have been shown to exhibit a greater response to fg-SRLs [17, 18]. As expected, also the expression of SSTR2 and SSTR5 seems to play a certain role in predicting the response to fg-SRLs [19–21]. More recently, we showed that acromegaly patients who exhibit a GH paradoxical increase (GH-Par) after an oral glucose tolerance test (OGTT)– i.e., about one-third of cases– had a better response to fg-SRLs [22]. As confirmed by other studies, these patients, presenting with higher IGF-1 levels at diagnosis, generally exhibit a milder phenotype– i.e., smaller and less invasive adenomas– compared to those with a conventional GH response after OGTT [22–24].

Regarding the outcome of PAS treatment, only a limited number of prognostic markers are currently available [25], linked to adenoma granularity and T2-weighted MRI signal intensity. Sparsely granulated adenomas– a pattern typically associated with a hyperintense signal– generally exhibit better responses to PAS treatment, although in some cases, tumor shrinkage was the only observed correlation [5].

In this study, we investigated whether the paradoxical GH response to OGTT can serve as a biomarker for both the biochemical/radiological response to PAS and as a predictive factor for adverse events.

## Materials and methods

### Patients

The data collection initiative was proposed during a meeting of the Italian Society of Endocrinology dedicated to hypothalamic and pituitary diseases in February 2023, and seven referral centers in Italy joined the initiative. The local ethics committees approved the study protocol (n° AOP1782, ref. 4834/AO/20), which was conducted in accordance with the Helsinki Declaration. Informed consent was obtained from each patient. Some patients, along with their clinical characteristics, have been previously described [26–30].

A total of 78 patients, enrolled in the study between 2013 and 2024 were considered for inclusion. However, data from the Oral Glucose Tolerance Test (OGTT) at the time of diagnosis were available for 59 patients, who were consequently included in the analysis. In addition, the inclusion criteria required that patients have undergone at least three consecutive months of PAS monotherapy and have been diagnosed with active acromegaly, in accordance with international criteria, including unsuppressed serum GH levels after OGTT, elevated IGF-1 levels at diagnosis, and MRI evidence of a pituitary adenoma or GH-positive adenoma at immunostaining. Patients who had undergone radiotherapy within ten years prior to the initiation of PAS were excluded from the study.

Patients’ clinical and hormonal data at diagnosis, before and after PAS treatment, along with radiological data were collected and analyzed retrospectively. GH, IGF-1, and prolactin levels were assessed at each center using commercially available laboratory kits. IGF-1 values were expressed as a percentage of the upper limit of normal (ULN) for age and gender. Additionally, preceding treatments to PAS, including fg-SRL usage (with details on maximum dosage and duration, co-administration with PEG and/or DA), surgical interventions (number of procedures), and radiotherapy, were also recorded. Radiological data, including adenoma’s maximum diameter, T2-weighted tumoral pattern (evaluated by comparing adenoma's mean T2 intensity to that of gray matter in the temporal cortex) and cavernous sinus invasion (CSI) were collected from gadolinium MRI scans at diagnosis. Immunohistochemical analysis using a standard approach was conducted to evaluate pituitary hormones, the Ki-67 proliferation index (classified as high or low based on ≤ 3% or > 3%), and the granulation pattern evaluated through cytokeratin staining. Glucose metabolism alterations (GMAs) were assessed at acromegaly diagnosis and before initiating any treatment. According to international criteria, GMAs included impaired fasting glucose (IFG: basal glucose 5.5– 7.0 mmol/L), impaired glucose tolerance (IGT: glucose levels 120 min after OGTT 7.8–11.1 mmol/L), and diabetes mellitus (DM: basal glucose ≥ 7 mmol/L or glucose levels 120 min after OGTT ≥ 11.1 mmol/L or HbA1c ≥ 48 mmol/mol, or use of anti-diabetic drugs).

All patients underwent OGTT for diagnostic purposes following a standard protocol [22]. Briefly, blood was sampled after an overnight fast and then at 30, 60, 90, and 120 min following oral glucose administration and GH, glucose, and insulin levels were recorded. Based on our earlier observations, GH-Par was defined as the glucose-induced GH response, with a peak-to-basal GH ratio ≥ 120% achieved within 90 min after glucose challenge [31].

After PAS treatment, data on adenoma shrinkage (≥ 20%), new-onset GMAs or significant worsening of DM (defined as persistent DM despite multimodal oral and injectable anti-diabetic treatment), and the status of ongoing PAS treatment at the final follow-up visit (or the reason for discontinuation) were recorded. Given the limited success observed with multimodal therapy in our patient cohort, responder status was assigned to patients whose IGF-1 levels were normalized or reduced by at least 30% at the last follow-up visit.

### Statistical analysis

Our study adheres to the STARD (standards for reporting diagnostic accuracy studies) criteria and follows the Strengthening the Reporting of Observational Studies in Epidemiology (STROBE) statement and guideline [32].

Continuous variables were presented using means and standard error of the mean (SEM) for normally distributed data, and medians with interquartile ranges (IQRs) for non-normally distributed data. The unpaired Student’s t-test or Mann–Whitney test was used for quantitative variables after assessing normality using the Kolmogorov–Smirnov Z test. Group comparisons for categorical variables were performed using the Fisher exact test. Data management and statistical analysis were conducted using R studio v 1.2.5033. A significance level of p < 0.05 was applied to all tests. All data analyzed in this study are stored in the data repositories of the University of Padova—Research Data UniPD (doi: 10.25430/researchdata.cab.unipd.it.00001165).

## Results

### Pre-PAS treatment clinical features

We collected data from a cohort of 59 patients with acromegaly, comprising 40 females (median age at diagnosis, 45.0; IQR 34.0 to 55.0 years) and 19 males (39.0; IQR 31.0 to 50.5 years), with one patient diagnosed with pediatric GH excess. All patients presented elevated IGF-1 levels at diagnosis (median 728.0; IQR 515.0 to 913.5 µg/L, 2.84, 2.05 to 3.69 ULN). In one case, GH excess was diagnosed during childhood. The median baseline maximum adenoma diameter, available for 51/59 patients, was 18 mm (IQR 13.0 to 26.0). The cohort predominantly consisted of large and invasive adenomas, resistant to standard therapy, requiring multimodal treatment combining surgery, radiotherapy, and fg-SRLs. Microadenomas were observed in only 6 patients, with three of them exhibiting CSI. Among the 45 macroadenomas, 37 also displayed CSI, resulting in an overall 82% incidence of invasive adenomas. Surgical intervention was performed once in 35 patients, twice in 7 patients, and three times in one case. Additionally, 10 patients underwent radiotherapy post-pituitary surgery, with 3 undergoing conventional and 7 undergoing stereotactic radiotherapy. Regarding fg-SRLs treatment, 59% of patients received LAN for durations ranging from 3 to 216 months, with a median of 28 months (IQR 15 to 60 months). This includes one patient receiving LAN 90 mg monthly, 29 patients on LAN 120 mg/month, and 3 patients on LAN 160 mg/month. The remaining 41% of patients received OCT, with one patient on OCT 10 mg/month, and 11 patients each on OCT 30 mg/monthly and OCT 40 mg/monthly. Treatment durations ranged from 4 to 31 months, with a median of 21 months (IQR 11 to 31 months). Data on fg-SRL type were unavailable in three cases. There was no significant difference in treatment duration and dose between OCT and LAN (p = 0.15). Combination therapy with fg-SRLs and PEG, DA, or a combination of PEG and DA was used in 14, 12, and 5 patients, respectively.

**Fig. 1:**
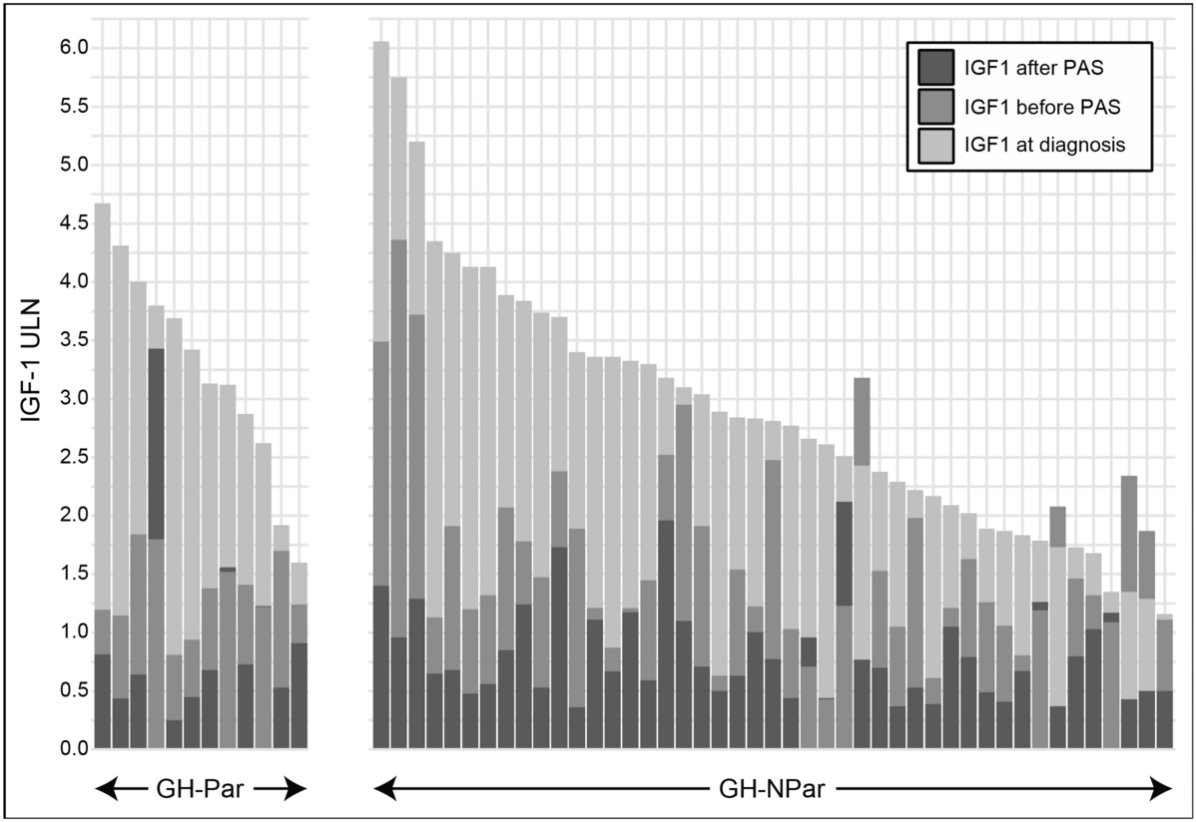
IGF-1 ULN levels in GH-Par and GH-NPar patients. The light gray bar represents IGF-1 ULN at diagnosis, the gray bar represents IGF-1 ULN before PAS, and the dark gray bar represents IGF-1 ULN after at least 3 months of PAS treatment. An overlap of the dark gray bar onto the gray one denotes a lack of positive response to PAS

Prior to initiating PAS treatment, IGF-1 levels remained elevated (median 368.0; IQR 298.0—483.0 µg/L, ULN 1.38; IQR 1.15 to 1.89 ULN). When comparing baseline IGF-1 levels with the biochemical control of acromegaly immediately before PAS therapy, the entire cohort exhibited a median reduction of −51% in IGF-1 levels (from 753 µg/L to 416 µg/L; p < 0.001). Notably, IGF-1 levels before PAS therapy were lower than those at diagnosis in 92% of patients (Fig. 1).

Moreover, in terms of comorbidities, 46% of patients presented with arterial hypertension, and 44% had GMAs.

### Response to PAS treatment

According to our definition of responsiveness to PAS 41 out of 57 patients were considered responders or partially responders to this drug.

Before PAS treatment acromegaly was not controlled (i.e., IGF-1 ULN > 1) in 86% of the cohort. For the remaining 14% of patients, acromegaly was adequately controlled with fg-SRLs alone or in combination with DA or PEG. However, the switch to PAS therapy was made for various reasons. Specifically, PAS was initiated in three patients to address headaches that were inadequately controlled by previous treatments. In four other cases, PAS monotherapy was introduced to simplify existing combination therapies. Additionally, for one patient who responded well to PEG but was unresponsive to fg-SRLs, PAS was selected due to the logistical challenges associated with daily subcutaneous PEG injections. PAS doses were 20, 40, and 60 mg/monthly injections in 2, 29, and 28 cases, respectively, administered for durations ranging from 3 to 144 months (median 24.0; IQR 12.0 to 45.5 months). Overall, the median reduction of IGF-1 ULN was −54.4% (IQR, −64% to −22%). PAS treatment significantly reduced final IGF-1 levels compared to levels before PAS, decreasing from a median of 368 µg/L (IQR, 298 to 483, 1.38 ULN; IQR 1.15 to 1.89) to 178 µg/L (IQR 146.5 to 276.5, 0.70 ULN; IQR to 0.50–1.07) (p < 0.001). As shown in Fig. 1, among patients with available pre-PAS IGF-1 ULN (n = 57), PAS did not result in further reduction of IGF-1 ULN levels in 8 cases (7 females and one male, 3 GH-Par and 5 GH-NPar). Among these, IGF-1 levels remained relatively stable in five patients (ULN increase ranging from 0 to 7%, mean 4% ULN, with a mean PAS duration of 24.8 months, ranging from 4 to 48 months). In contrast, the other three patients experienced an increase in IGF-1 ULN levels (ranging from + 35% to + 91%). These particularly resistant cases included one GH-Par and two GH-NPar, all presenting with macroadenomas and CSI, with one patient having undergone RT. In the remaining 49/57 patients, a further reduction in IGF-1 ULN of −59% (IQR −64.8% to −32%) was observed. A reduction of at least 30% in IGF-1 ULN was reported in 41/49 patients, among whom IGF-1 normalization was achieved in 37/41 subjects. Specifically, within the group of 41 patients, IGF-1 levels decreased from a median of 398 µg/L to 164 µg/L after PAS (p < 0.001), corresponding to a reduction from 1.47 to 0.64 times the ULN (p < 0.001).

Among patients with available imaging assessment before and after PAS treatment (n = 39), five cases exhibited a ≥ 20% reduction in the size of the GH-sec PAs after an average therapy duration of 68 months. All patients received PAS 60 mg/monthly.

PAS was discontinued in seven patients due to the new-onset GMAs or worsening of DM. Additionally, PAS was discontinued in further nine patients due to reasons such as uncontrolled acromegaly, other side effects (e.g., bradycardia and hospitalization for Takotsubo syndrome), or new surgical attempts.

Regarding metabolic comorbidities, after PAS therapy, our cohort exhibited a general worsening of the glucose metabolism profile. Specifically, the median fasting glucose increased from 5.45 mmol/L (IQR 5.1 to 6.6) to of 6.5 mmol/L (IQR 5.85 to 8.7) (p < 0.001), and HbA1c rose from 39 mmol/mol (IQR 37 to 42) to 45 mmol/mol (IQR 41 to 51.9) (p < 0.001). The prevalence of GMAs increased from 44 to 80% after PAS treatment, (p = 0.005). No significant differences were observed in terms of insulin levels, total cholesterol, HDL cholesterol, LDL cholesterol, triglycerides or systolic and diastolic pressure in the entire cohort following PAS treatment.

### Comparison between GH-Par and GH-NPar acromegaly

**Fig. 2:**
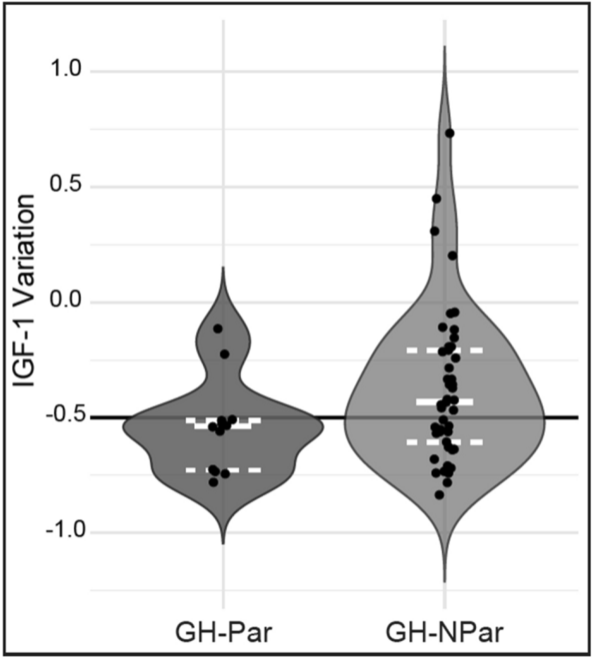
Violin plot illustrating the variation in IGF-1 ULN levels at diagnosis and before PAS, organized for patients with GH-Par and GH-NPar. The solid white line indicates the median, the dashed white line represents the interquartile range, and the black line marks the 50% reduction threshold in IGF-1 levels

According to our definition of paradoxical response of GH to OGTT, thirteen patients (22%) exhibited GH-Par, while the remaining forty-six (78%) were categorized as GH-NPar. At diagnosis, hormone levels and adenoma features were similar in GH-Par and GH-NPar patients (Table 1). Although IGF-1 ULN levels did not significantly differ between GH-Par and GH-NPar patients at diagnosis or before PAS initiation, GH-Par patients tended to experience a more pronounced reduction in IGF-1 levels during treatments preceding PAS (Fig. 2, Table 2). Notably, 83% of GH-Par patients, compared to only 42% of GH-NPar patients, showed a decrease of at least 50% from the baseline IGF-1 ULN after pre-PAS treatment (p = 0.02).

**Table 1:** Endocrine and clinical features of acromegaly patients at the time of diagnosis, stratified based on their GH profile at OGTT

	GH-Par n = 13	GH-NPar n = 46	P value
Gender (M/F)	2/11	17/29	0.19
Median age at diagnosis, years	49 (40–55)	39 (33–50.8)	0.21
Hyper-prolactinemia (yes/no)	2/11	12/32	0.48
Median IGF-1 at diagnosis, µg/L	823 (662–890)	701 (498.2–955.5)	0.53
Median IGF-1 ULN at diagnosis	3.2 (2.6–3.8)	2.79 (1.923–3.4)	0.20
Median GH at diagnosis, µg/L	6.1 (4.2–11.4)	8.2 (5.5–19.9)	0.12
Median Glucose at diagnosis, mmol/L	5.4 (4.9–6.1)	5.3 (4.9–5.8)	0.57
Median Glucose 120 min OGTT, mmol/L	7 (5.7–8)	6.8 (6.2–8.3)	0.93
Median Insulin at diagnosis, mU/L	26.4 (6.1–33.9)	11.5 (6.6–16)	0.42
Median Insulin peak OGTT, mU/L	110.0 (36.6–195)	149.0 (58.4–195.8)	0.64
Median maximal diameter, mm^§^	16 (10–34)	18.0 (13.3–24.8)	0.90
CSI (yes/no)^§^	7/5	35/8	0.13
T2 hypointense signal (yes/no)	0/5	4/13	0.53
Pure GH adenoma (yes/no)	6/2	21/11	1.00
Average number of surgeries (range)	0.85 (0–2)	0.89 (0–3)	0.88
Ki-67 index, low/high^§^	6/2	16/5	1.00
Median duration of treatment with fg-SRLs, months	28 (11.8–63.8)	24 (12–42)	0.56
Normalized dose fg-SRLs*	1 (1–1.2)	1 (1–1.1)	0.90

Median values are expressed with IQRs in parentheses

M: male, F: female; CSI: cavernous sinus invasion; ULN: upper limit of normal; fg-SRL: first-generation somatostatin receptor ligands; OGTT: oral glucose tolerance test

^§^This data referred to the first surgery

*The normalization is achieved by dividing the SRL dose by 30 mg (for octreotide) or 120 mg (for lanreotide)

**Table 2:** Endocrine and clinical features of acromegaly patients, stratified based on their GH profile at OGTT immediately before and after PAS treatment

	GH-Pa**r** N = 13	GH-NPar N = 46	P value
Type of treatment before PAS fg-SRL alone/ + PEG/ + DA/ + DA and PEG	7/3/1/2	21/11/11/3	0.50
Median IGF-1 levels before PAS, µg/L	317.5 (263.5 to 364)	379 (300 to 561)	0.09
Median IGF-1 ULN before PAS	1.3 (1.2 to 1.6)	1.45 (1.1 to 2)	0.41
Median IGF-1 ULN reduction due to treatment preceding PAS	−53.7% (−72.7% to −51.2%)	−43.3% (−60.6% to −20.1%)	0.11
Reduction of IGF-1 ULN due to treatments preceding PAS, > 50%/ ≤ 50%, %	83%/17%	42%/58%	0.02
Median maximal dose PAS, mg/monthly	40 (40 to 60)	50 (40 to 60)	0.59
Median duration of PAS treatment, months	16 (8 to 33)	24.5 (13.5 to 56.5)	0.22
IGF-1 after PAS, µg/L	162 (146 to 224)	183 (148.8 to 280.2)	0.44
IGF-1 ULN after PAS	0.7 (0.5 to 0.9)	0.7 (0.5 to 1.1)	0.93
IGF-1 ULN reduction with PAS, %	−49.5% (−62.8% to −20%)	−57.3% (−64% to −22%)	0.55
PAS responsiveness (yes/no)	9/3	32/13	1.00
Median GH after PAS, µg/L	1.2 (0.0 to 1.9)	0.8 (0.5 to 1.9)	0.80
Radiotherapy (yes/no)	2/11	8/37	1.00
PAS-induced ≥ 20% adenoma shrinkage (yes/no)	0/7	5/28	0.56
Ongoing PAS therapy (yes/no)	6/6	27/10	0.17

Median values are expressed with IQRs in parentheses

PAS: pasireotide, fg-SRL: first-generation somatostatin receptor ligands, DA: Dopamine Agonist, PEG: Pegvisomant

The duration and dosage of PAS therapy, as well as its effectiveness in reducing IGF-1 levels, were similar between the GH-Par and GH-NPar groups (Table 2).

With respect to metabolic parameters, median fasting glucose and HbA1c levels were comparable between the two groups both before and after PAS treatment (Table 3). However, there was a notable worsening of fasting glucose after therapy in both groups. Specifically, in GH-Par patients, fasting glucose increased from a median of 6.13 mmol/L (IQR 5 to 6.85) to 7.35 mmol/L (IQR 6.12 to 10.9) (p = 0.006). In GH-NPar patients, fasting glucose increased from 5.32 mmol/L (IQR 5.1 to 6.28) to 6.3 mmol/L (IQR 5.7 to 8) (p < 0.001). HbA1c values also worsened in both groups, with GH-Par patients experiencing an increase from 38 mmol/mol (IQR 33.5 to 42) to 45.5 mmol/mol (IQR 40 to 54) (p = 0.004), and GH-Par patients showing an increase from 39 mmol/mol (IQR 37 to 41.5) to 45 mmol/mol (IQR 41 to 51) (p < 0.001). The prevalence of GMAs was higher in the GH-Par group than in the GH-NPar group before PAS treatment (69% *vs* 37%, p = 0.038). Although an increase in GMAs frequency was observed in both groups post-PAS, a statistically significant rise occurred only in the GH-NPar group, from 17/46 to 37/46 (p = 0.014). In contrast, the GH-Par group showed no significant variation (9/13 to 10/13, p = 1).

**Table 3:** Biochemical features of GH-Par and GH-NPar patients both compared before and after PAS treatment

	Baseline	Post PAS	P value^*^
Median fasting glucose GH-Par (mmol/l)	6.13 (5–6.8)	7.35 (6.1–10.9)	**0.006**
Median fasting glucose GH-NPar (mmol/l)	5.3 (5.1–6.3)	6.3 (5.7–8)	**0.001**
P value^#^	0.46	0.23	
Median insulin GH-Par (mIU/l)	24 (8–126.5)	7.6 (4.5–12.7)	0.35
Median insulin GH-NPar (mIU/l)	10.7 (5.9–25)	15.5 (9.1–33)	0.97
P value^#^	0.16	0.13	
Median HbA1c GH-Par (mmol/mol)	38 (33.5–42)	45.5 (40–54)	**0.004**
Median HbA1c GH-NPar (mmol/mol)	39 (37–41.5)	45 (41–51)	**0.001**
P value^#^	0.50	0.44	
Median SAP GH-Par (mmHg)	140 (120–160)	130 (127.5–140)	0.74
Median SAP GH-NPar (mmHg)	120 (115–135)	120 (115–130)	0.54
P value^#^	**0.038**	**0.039**	
Median DAP GH-Par (mmHg)	80 (80–87.5)	88 (77.5–90.5)	0.35
Median DAP GH-NPar (mmHg)	80 (70–85)	80 (70–90)	0.90
P value^#^	0.28	0.13	
Median total cholesterol GH-Par (mmol/l)	5 (4.5–6)	4.8 (3.3–5)	0.48
Median total cholesterol GH-NPar (mmol/l)	5.1 (4.8–5.9)	5.25 (4.5–5.7)	0.76
P value^#^	0.83	0.11	
Median HDL GH-Par (mmol/l)	1.5 (1.2–1.9)	1.5 (1.2–1.7)	0.99
Median HDL GH-NPar (mmol/l)	1.5 (1.2–1.9)	1.5 (1.2–1.8)	0.99
P value^#^	0.71	0.15	
Median triglycerides GH-Par (mmol/l)	1.2 (0.8–1.8)	1.1 (0.9–1.6)	0.93
Median triglycerides GH-NPar (mmol/l)	1 (0.8–1.9)	1.2 (0.8–1.6)	0.45
P value^#^	0.44	0.88	
Median LDL GH-Par (mmol/l)	3.0 (2.2–3.5)	2.9 (1.1–3.2)	0.43
Median LDL GH-NPar (mmol/l)	3.5 (2.5–4)	3.3 (2.4–4)	0.56
P value^#^	0.37	0.17	
GMAs GH-Par (yes/no)	9/4	10/3	1.00^§^
	9 yes = 3 DM, 5 IFG, 1 IGT	10 yes = 8 DM, 1 IFG, 1 IGT	
GMAs GH-NPar (yes/no)	17/29	37/9	** < 0.001**^**§**^
	*17 yes* = *4 DM, 8 IFG, 5 IGT*	*37 yes* = *21 DM, 10 IFG, 6 IGT*	
P value^#^	**0.038**	0.72	

Median values are expressed with IQRs in parentheses

SAP: systolic arterial pressure, DAP: diastolic arterial pressure, HDL: High-Density Lipoprotein, LDL: Low-Density Lipoprotein, DM: diabetes mellitus, IFG: impaired fasting glucose, IGT: impaired glucose tolerance

^#^Refers to the comparison between GH-Par and GH-NPar groups

^*^Refers to the comparison between baseline and post-PAS values

^§^McNemar test

GH-Par subjects exhibited higher median systolic blood pressure compared to GH-NPar patients, both before PAS (140 mmHg [IQR 120 to 160] vs 120 mmHg [IQR 115 to 135], p = 0.038) and after PAS (130 mmHg [IQR 127.5 to 140] vs 120 mmHg [IQR 115 to 130], p = 0.039). No significant differences were observed in terms of insulin levels, total cholesterol, HDL and LDL cholesterol, or triglycerides between the two groups before and after PAS treatment.

## Discussion

This study aimed to further explore the clinical heterogeneity associated with the paradoxical rise of GH in response to a glucose challenge, specifically its ability to predict outcomes in patients undergoing PAS treatment, mirroring findings with fg-SRLs [22–24]. The acromegaly patients from seven Italian clinics were not selected consecutively but rather with specific selection biases, resulting in a cohort with a high prevalence of aggressive forms (e.g., anatomical invasion, rapid growth, resistance to standard treatments [33]). This is expected given the criteria for initiating PAS therapy, which include resistance to standard-high doses of fg-SRLs and/or persistence after transsphenoidal surgery. This bias likely also explains the higher proportion of female patients, as female patients, particularly postmenopausal women, often have larger, more aggressive adenomas, lower surgical remission rates [34], and a higher standardized mortality ratio [35]. In comparison to previously analyzed datasets employing a similar definition of GH-Par [22, 31], this study exhibits a lower prevalence of GH-Par (22% *vs* ~ 40%). Since the use of PAS is inherently linked to resistant acromegaly and considering that the GH-Par is associated with an increased fg-SRL responsiveness [22–24], a reduced prevalence of paradoxical cases in our cohort is not unexpected.

Interestingly, even in this study, a somewhat less severe phenotype in paradoxical patients compared to non-paradoxical ones emerges, albeit not prominently. Specifically, at the beginning of PAS treatment, the GH-Par group exhibited a more favorable response to preceding therapies than the GH-NPar group, with a greater reduction in IGF-1 ULN values observed in these cases. This is likely due to their greater responsiveness to previous treatments with fg-SRLs [22, 24, 36]. However, what does not emerge from analyzing this cohort is a differential responsiveness to PAS therapy based on patients’ GH profile during OGTT. In this series, indeed, the rate of IGF-1 normalization with PAS was substantial and consistent with other real-life series^5^, but remarkably similar in GH-Par and GH-NPar groups, showing a significant response in 83% and 89% of cases, respectively. In 2021, Atquet et al. reported the use of PAS in patients with and without paradoxical GH response to OGTT [24]. While the authors noted a better response of GH-Par patients to SRLs, their study combined data from both first- and second-generation SRLs, making it challenging to directly compare with our study's findings. Additionally, Atquet et al. defined treatment resistance based on a PAS dose exceeding 20 mg monthly, a criterion inconsistent with our experience, where higher PAS doses (often 40 mg/monthly or 60 mg/monthly) were common in nearly all cases [24].

Beyond the potential influence of heterogeneity in preceding treatments, the reasons why the observed differences between GH-Par and GH-NPar under fg-SRL treatment do not emerge during PAS therapy are not immediately clear. The presence of various factors influencing responsiveness to PAS and fg-SRLs is unequivocally established, which include the granulation pattern and the differential expression pattern of SSTR [21]. As far as we know, there is no available data on SSTR expression profiles in GH-Par and GH-NPar. Nevertheless, Faria et al. recently demonstrated that GH-sec PAs positive and negative for the expression of the glucose-dependent insulinotropic polypeptide receptor (GIPR) exhibit a similar pattern of SSTR2 and SSTR5 expression [37]. While not all GH-Par are GIPR^+^ and GH-NPar GIPR^−^, the correlation between these two parameters tends to exceed 80% [38, 39], suggesting that the differences in the SSTR profiles between GH-Par and GH-NPar likely does not play a key role in justifying their divergent response to therapy with fg-SRL and PAS. On the other hand, a well-established association exists between paradoxical GH response to OGTT and activation of the GIP/GIPR axis in GH-sec PAs [38–40], and the influence of SRLs on incretin secretion [41] is widely recognized. The question of whether PAS and fg-SRLs may elicit distinct effects on GIP secretion, and consequently, on its stimulatory effect in GIPR^+^ GH-sec PA but not in GIPR^−^ ones, remains an open question.

One of the most significant side effects induced by PAS is the deterioration of glucose metabolism [42], leading to the onset of new GMAs. These abnormalities range from impaired post-prandial glucose levels to overt diabetes mellitus. In patients with Cushing’s disease, the onset of GMAs can be predicted after a single dose of subcutaneous PAS [43]. However, with prolonged treatment, PAS has been observed to reduce insulin secretion and improve insulin sensitivity, attributed to the reduction in cortisol levels [44]. In acromegaly, predictive factors for PAS-induced diabetes seems to be associated with the patient's age, along with a history of diabetes, hypertension, and dyslipidemia [45]. Additionally, Corica et al. recently identified older age at acromegaly diagnosis and before PAS initiation, as well as female sex, as possible predictors of the worsened glucose metabolism after PAS treatment [27]. In accordance with prior findings [24, 31] the current study also revealed significant differences in metabolic complications between GH-Par and GH-NPar patients. Specifically, the incidence of GMAs before PAS therapy was nearly double in patients with GH-Par compared to those with stable or reduced GH levels after OGTT (69% *vs* 37%). Conversely, while PAS treatment did not significantly alter the prevalence of GMAs in GH-Par patients, the change was substantial in GH-NPar cases, where a marked worsening of the metabolic profile occurred. Although one might conclude that GH-Par patients are "less sensitive" to PAS-induced metabolic complications, it is likely more accurate to state that, for most of these patients by the time they are considered for PAS treatment, their metabolic condition may be already somewhat compromised, which could explain they do not experience further deterioration. On the other hand, a negative impact of PAS on the metabolic control of acromegalic GH-NPar patients is conceivable. Therefore, while there is no clear evidence to suggest that the OGTT GH profile should be incorporated into a model for predicting PAS-induced GMAs, it may still provide some guidance for therapeutic decisions.

In regarding to the impact of OGTT GH profiles on GMA prevalence, it is reasonable to hypothesize that this is related to IGF-1 levels. Indeed, higher IGF-1 levels at diagnosis can predict the development of GMAs in acromegaly patients [46–48], and GH-Par patients typically have higher IGF-1 levels compared to GH-NPar patients [22, 49]. Given the effects that IGF-1 seems to have on GIP [38], the notion of a self-reinforcing system in GH-Par patients is not implausible.

Beyond the reasons underlying this phenomenon, our data support the notion that the effects of PAS on glucose metabolism should be carefully monitored in all patients with acromegaly. In fact, although novel PAS-induced GMA is more frequent in the GH-NPar patients, an increase in diabetes following PAS is observed in both GH-Par and GH-NPar groups. In clinical practice, if PAS treatment is considered appropriate, proactive management of PAS-induced hyperglycemia is crucial [49]. Both DPP-4 inhibitors and GLP-1 receptor agonists have been shown to be effective treatments.

This study, which further investigates the prognostic significance of OGTT [22, 49], which may overcome its diagnostic utility [50, 51], is however not without limitations. The retrospective and multicenter design, conducted in a real-life setting, introduces variability in the approaches to resistant acromegaly, which is the main limitations of our study. However, the number of patients enrolled may mitigate these limitations to some extent. In a heterogeneous cohort with aggressive manifestations of a rare disease such as acromegaly, any significant (or near-significant) difference observed may hold greater value than would be the case in a prospective study. We addressed variations in laboratory assays and IGF-1 ranges by considering both IGF-1 concentration and ULN. Although a prospective study could provide additional evidence, its implementation in clinical practice is currently impractical. In addition, we recognize the enrollment bias, as most of patients treated with pasireotide were unresponsive to first-generation SRLs, which may have affected the outcomes. However, since PAS is used exclusively as a second-line therapy, it remains to challenging to assess its effect in naive patients.

In conclusion, our findings confirm the effectiveness of PAS in controlling acromegaly activity in resistant patients, regardless of their GH profile at OGTT. We further confirm that GH-Par patients, even when not fully responsive to pre-PAS therapies, exhibit a more substantial reduction in IGF-1 levels in comparison to GH-NPar patients. Finally, GH-Par patients typically present with a worse metabolic profile when being considered for PAS, although this profile does not deteriorate further with PAS treatment, unlike in GH-NPar patients.

## Data Availability

All data generated or analyzed during this study are included in this published article or the data repositories in the public available website of the University of Padova (doi: 10.25430/researchdata.cab.unipd.it.00001165).
